# The Metropolitan Asylums Board and Their Staff

**Published:** 1913-05-03

**Authors:** 


### The Metropolitan Asylums Board and their Staff.

THE SALARIES UNDER THE APPROVED SCHEME.

At their last meeting the Metropolitan Asylums Board considered exhaustive reports embodying the results of a special inquiry into the pay and certain of the conditions of service of their staff. The inquiry originated in a petition presented direct to the Board by a large body of their mental-hospital employees, and the proposals were prepared by a special sub-committee who considered this petition. At the same time representations made by the Municipal Employees' Association were considered, and it was decided that the Board's Finance Committee should, as far as possible, apply to the other branches of the Board's service in which officers of the same grades were employed the proposals approved for the mental-hospital service. The salaries of the nurses in the infectious hospitals and of a few other grades, having been revised quite recently, were not dealt with in the present scheme.

The following are the chief features of the approved scheme, the cost of which to the Board is estimated to reach £20,000 per annum :—

(1) A revised scale of salaries and wages has been approved, and will come into force from June 1 next.

(2) A revised scale of valuation of emoluments has been approved, and this scale will apply in future both for superannuation purposes and for living-out allowances. The majority of employees consider that a high valuation for superannuation purposes is in the Board's interest, and the old valuation has therefore, in some instances, been reduced, while the living-out allowances have in some instances been increased. This new valuation must be accepted by all employees who wish to be placed on the revised salaries and wages scale; but any who elect to have their emoluments valued at the existing rates may do so by remaining on the existing scale of pay.

(3) The payment of an allowance in lieu of board will be made to employees boarded or partially boarded when absent from the institution on duly authorised annual leave.

(4) Worn-out uniforms will be retained by employees subject (i) to the official buttons from the male staff uniform being given up, and (ii) to the last issue of uniform being given up by employees with less than six months' service since its issue.

(5) A proportion of annual leave, according to a fixed scale, will be granted to all employees entitled to annual leave, but with less than twelve months' service at the beginning of the holiday season in any year.

(6) The hours of duty of asylum attendants and nurses will be those proposed in the Asylums Officers (Employment, Pension, and Superannuation) Bill, and unanimously approved by a Select Committee of the House of Commons on which all parties were represented—viz., seventy per week on day duty and sixty per week on night duty, calculated over a period of two weeks, including fire, reserve or emergency duty, but excluding any fixed period for meals or rest.

(7) No reduction will be made in future in the annual leave of attendants, and nurses with less than two years' service, and an increase has been made in the annual leave of charge and deputy-charge attendants.

(8) Special long-service pay will be given to mental-hospital attendants and nurses after five and ten years' service respectively.

(9) All laundrymaids will have the option of living out, and of receiving a monetary allowance in lieu of the emoluments—board, lodging, and washing.

